# Metabolomic Profile of Hepatitis C Virus-Infected Hepatocytes

**DOI:** 10.1371/journal.pone.0023641

**Published:** 2011-08-11

**Authors:** Barbara Roe, Elizabeth Kensicki, Robert Mohney, William W. Hall

**Affiliations:** 1 Centre for Research in Infectious Diseases, School of Medicine and Medical Science, University College Dublin, Belfield, Dublin, Ireland; 2 Metabolon Inc., Durham, North Carolina, United States of America; Duke University, United States of America

## Abstract

Hepatitis C virus (HCV) is capable of disrupting different facets of lipid metabolism and lipids have been shown to play a crucial role in the viral life cycle. The aim of this study was to examine the effect HCV infection has on the hepatocyte metabolome. Huh-7.5 cells were infected using virus produced by the HCV J6/JFH1 cell culture system and cells were harvested 24, 48, and 72-hours following infection. Metabolic profiling was performed using a non-targeted multiple platform methodology combining ultrahigh performance liquid chromatography/tandem mass spectrometry (UHPLC/MS/MS^2^) and gas chromatography/mass spectrometry (GC/MS). There was a significant increase in a number of metabolites involved in nucleotide synthesis and RNA replication during early HCV infection. NAD levels were also significantly increased along with several amino acids. A number of lipid metabolic pathways were disrupted by HCV infection, resulting in an increase in cholesterol and sphingolipid levels, altered phospholipid metabolism and a possible disruption in mitochondrial fatty acid transport. Fluctuations in 5′-methylthioadenosine levels were also noted, along with alterations in the glutathione synthesis pathway. These results highlight a number of previously unreported metabolic interactions and give a more in depth insight into the effect HCV has on host cell biochemical processes.

## Introduction

Hepatitis C virus (HCV) is a leading cause of liver disease and transplantation worldwide and is a major burden on public health [1]. According to WHO estimates, the global prevalence of HCV is approximately 2%, representing 130 million people infected worldwide [2]. Approximately 50–80% of individuals infected with HCV become chronically infected; of these 10–20% will develop liver cirrhosis and up to 5% of patients with HCV-related cirrhosis will develop hepatocellular carcinoma (HCC) [3]. There is no vaccine against HCV available and current treatment response rates are sub-optimal. At present, the standard treatment is a 24–48 week course with pegylated interferon alfa and ribavirin, resulting in only 40–50% of genotype 1-infected patients achieving a sustained virological response [4], [5].

HCV replication causes dramatic changes within infected hepatocytes including the disruption of different aspects of lipid metabolism. Lipids have also been shown to play important roles in the viral life cycle and pathogenesis of infection [6]. Viral entry is mediated in part through the use of lipoprotein receptors [7], [8] and HCV virions have been shown to circulate bound to lipoproteins in the serum of infected patients [9]. There is also evidence to suggest that HCV may use the VLDL assembly and secretion pathway for maturation and secretion of viral particles [10], [11]. Cholesterol and sphingolipids are important for virion maturation and infectivity, as cholesterol-depleted or sphingomyelin-hydrolysed virus negatively impact infectivity [12]. An increase in the lipid content within hepatocytes can result in liver steatosis; this is a prominent histological phenotype of HCV infection and has been associated with progression to liver fibrosis [13]. Although there are many published reports documenting the relationship between HCV and lipid metabolism, there is limited information available on the impact of HCV infection on global metabolism. One recent report demonstrated how HCV infection exploits other areas of metabolism; HCV was shown to disrupt normal metabolic homeostasis incurring a shift from energy consuming to energy conserving activities over time [14]. The development of the JFH1-based cell culture system [15], [16], [17] has provided opportunities to study global metabolism in more detail.

Deciphering the ways in which HCV can disrupt metabolic pathways for viral replication represents an important area for future therapeutic intervention. Although various genomic [18], [19], proteomic [14], [19], and lipidomic [14] analyses have been performed, comprehensive metabolomic studies have yet to be reported. Changes in protein expression levels may not exert substantial effects on the flux through metabolic pathways. However, they can dramatically affect the concentration of intermediary metabolites. As a result, measuring metabolite concentrations can depict the activities of metabolic pathways more accurately than quantifying the relevant enzymes or mRNAs encoding them [20], [21]. The metabolome directly influences the cell phenotype, more so than transcripts or proteins, so performing metabolomic analysis could offer a distinct advantage when trying to decipher disease pathogenesis. The aim of this study was to examine the effect HCV infection has on the hepatocyte metabolome, by comparing global biochemical profiles between HCV-infected and uninfected Huh-7.5 cells at different time points following infection.

## Methods

### Cell culture and in vitro transcription

The human hepatoma cell line, Huh-7.5, and the J6/JFH1 strain of HCV were kindly provided by Dr. Charles M. Rice (Rockefeller University, New York). Cells were maintained in DMEM containing sodium pyruvate (Invitrogen, Paisley, UK) and supplemented with 1% Pen/Strep, 1% non-essential amino acids, and 10% fetal bovine serum (Invitrogen). *In vitro* transcribed RNA was prepared as previously described with minor modifications [15].

### Transfection and infection of Huh-7.5 cells

To generate virus stocks for infection experiments, RNA was transfected into Huh-7.5 cells using Lipofectamine 2000 reagent (Invitrogen). Cell culture supernatants were harvested after 4–8 days and viral titres were determined by 50% tissue culture infectious dose (TCID_50_) as previously described [15]. For infection experiments (n = 3), cells were plated in 100 mm tissue culture dishes at a density of 1×10^6^ cells/plate and inoculated with conditioned media or virus-containing supernatant at a multiplicity of infection of 0.7 for 8 hours. Following initial infection, the supernatant was replaced with fresh media and incubated until harvest at 24, 48, or 72 hours post-infection.

### Sample Preparation

A non-targeted metabolic profiling platform was employed combining three independent platforms: ultrahigh performance liquid chromatography/tandem mass spectrometry (UHPLC/MS/MS^2^) optimized for detection of basic species, UHPLC/MS/MS^2^ optimized for detection of acidic species, and gas chromatography/mass spectrometry (GC/MS). Samples were processed as previously described [22], [23]. In brief, using an automated liquid handler (Hamilton LabStar, Salt Lake City, UT), protein was precipitated from homogenized cells with methanol that contained four standards to report on extraction efficiency. The resulting supernatant was split into equal aliquots, which were then dried under nitrogen and vacuum-desiccated for analysis on the three platforms. For the two UHLC/MS/MS^2^ analyses, aliquots were reconstituted in 0.1% formic acid (acidic conditions) or 6.5 mM ammonium bicarbonate, pH 8 (basic conditions). For the GC/MS analysis, aliquots were derivatized using equal parts solvent mixture acetonitrile:dichloro-methane:cyclohexane (5∶4∶1) and bistrimethyl-silyl-trifluoroacetamide with 5% triethylamine at 60°C for one hour. In addition, three types of controls were analyzed in concert with the experimental samples: samples generated from pooled experimental samples served as technical replicates throughout the data set, extracted water samples served as process blanks, and a cocktail of standards spiked into every analyzed sample allowed instrument performance monitoring.

### Metabolic Profiling

For UHPLC/MS/MS^2^ analysis, aliquots were separated using a Waters Acquity UHPLC (Waters, Millford, MA) and analyzed using an LTQ mass spectrometer (Thermo Fisher Scientific, Inc., Waltham, MA), which consisted of an electrospray ionization source and linear ion-trap mass analyzer. The MS instrument scanned 99–1000 *m/z* and alternated between MS and MS^2^ scans using dynamic exclusion with approximately 6 scans per second. Derivatized samples for GC/MS were separated on a 5% phenyldimethyl silicone column with helium as the carrier gas and a temperature ramp from 60°C to 340°C and then analyzed on a Thermo-Finnigan Trace DSQ MS (Thermo Fisher Scientific, Inc.) operated at unit mass resolving power with electron impact ionization and a 50–750 atomic mass unit scan range.

### Metabolite Identification and Data Analysis

Metabolites were identified by automated comparison of the ion features in the experimental samples to a reference library of chemical standard entries that included retention time, molecular weight (*m/z*), preferred adducts, and in-source fragments as well as associated MS/MS^2^ spectra and curated by visual inspection for quality control using software developed at Metabolon Inc. [24]. This method permits both qualitative identification and relative quantification of a broad range of small molecules. For statistical analyses and data display purposes, missing relative quantification values (if any) were imputed with the observed minimum for that particular metabolite. Statistical analysis of log-transformed data was performed using the “R” software package [25] and Welch's two-sample t-tests were used to identify metabolites that differed significantly between HCV-infected and uninfected cells at each time point examined. A p-value of <0.05 was considered statistically significant and multiple comparisons were accounted for by estimating the false discovery rate using q-values.

## Results

To examine the effect of HCV infection on different metabolic pathways, global biochemical profiles were compared between HCV-infected and uninfected Huh-7.5 cells at different time points following infection – 24, 48, and 72 hours post-infection. A total of 250 metabolites were detected and quantified, of which 73 were differentially regulated (Table S1). Data sets were also compared between time points in order to examine the natural fluctuations of metabolites over time (Table S2). Table 1 shows the breakdown of the differentially regulated metabolites at the different time points following HCV infection. In total, 40 metabolites were significantly increased, 31 decreased and 2 metabolites both increased and decreased (at different time points) relative to control (Table S1). Based on the findings of this study, it would appear that many different metabolic pathways are disrupted as a result of HCV infection, most notably fatty acid, phospholipid, amino acid, nucleotide and methylthioadenosine metabolism.

**Table 1:** Differentially regulated metabolites at different time points following HCV infection.

Welch's Two Sample T-Test	24 h	48 h	72 h
Total number of metabolites with p<0.05	25	27	42
Number of metabolites significantly increased	23	8	24
Number of metabolites significantly decreased	2	19	18

### Increased Metabolic Demands during Early Infection

Elevated metabolism and altered energy status were associated with early HCV infection. At the 24-hour time point, several intermediates involved in or derived from the pentose phosphate pathway, such as sedoheptulose-7-phosphate and xylonate, and a number of amino acids were significantly elevated in HCV-infected cells (Table 2). An increase in a number of metabolites involved in nucleotide metabolism and RNA replication was also noted; levels of adenosine, inosine, GMP and UMP were significantly increased at the 24-hour time point compared to control (Table 2) with moderate increases in AMP, adenylosuccinate and uridine diphosphate. NAD+ levels were also significantly elevated (p = 0.02) reflecting an altered energy status within HCV-infected cells.

**Table 2:** Increase in the levels of biosynthetic metabolites at the 24-hour time point post-HCV infection.

Metabolic Pathway	Metabolite	Mock-infected	HCV-infected	P value
Amino acid	Alanine	1.165±0.046	1.341±0.036	0.009
	Isoleucine	2.039±0.109	2.372±0.091	0.019
	Serine	1.709±0.033	1.851±0.056	0.026
	Tryptophan	1.821±0.040	2.064±0.001	0.010
	Tyrosine	1.807±0.055	2.144±0.029	0.004
RNA synthesis	Adenosine	0.969±0.044	1.176±0.057	0.008
	Inosine	0.962±0.110	1.252±0.124	0.027
	GMP	1.002±0.005	1.143±0.054	0.041
	UMP	1.152±0.034	1.256±0.023	0.020
Pentose phosphate pathway	Sedoheptulose-7-phosphate	0.606±0.127	1.158±0.142	0.013
	Xylonate	1.100±0.0860	1.657±0.250	0.03

Data displayed represent the mean scaled ion intensity values ± standard deviation.

### Altered Phospholipid Metabolism

**Figure 1:**
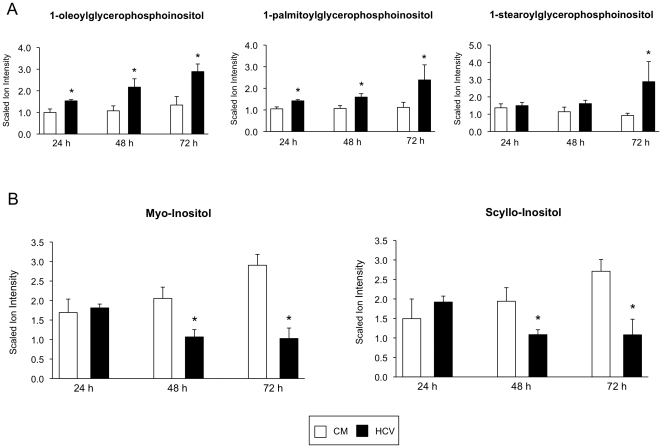
Altered lysophospholipid metabolism. Bar charts show the scaled ion intensity for (**A**) inositol-based lysophospholipids, and (**B**) Inositol-based lysophospholipid metabolites in HCV-infected cells (black bars) and mock-infected cells (white bars) 24, 48, and 72 hours post-infection. An asterisk (*) denotes a statistically significant difference with p<0.05. Data are presented as the mean of three separate experiments with error bars representing standard deviation. CM, conditioned media.

Phospholipid hydrolysis occurs through the actions of the phospholipase A_2_ enzyme family, and results in the generation of free fatty acids along with lysophospholipids (LPLs) [26]. A number of LPLs, most notably the inositol-based LPLs, and fatty acids were significantly increased at all time points following HCV infection (p<0.05; Figure 1A). Myo-inositol and scyllo-inositol act as both precursors and catabolites in LPL metabolism. A significant decrease in the levels of myo-inositol and scyllo-inositol at the 48- and 72-hour time points (p<0.05; Figure 1B), along with an increase in inositol-based LPLs suggest that inositol-based LPL catabolism may be impeded. At the 72-hour time point, a significant increase in the choline-based lysophospholipid, 2-palmitoylglycerophosphocholine was noted (p = 0.03). There was also a moderate increase in a number of other choline based LPLs and the downstream LPL catabolite, glycerophosphocholine.

A number of intermediates involved in phosphatidylcholine (PC) synthesis were elevated during HCV infection; CDP-choline and phosphorylcholine levels were significantly increased in HCV-infected cells at the 24 and 72-hour time points, respectively (Table S1). Synthesis of PC can also occur through the methylation of another phospholipid, phosphatidylethanolamine, by S-adenosylmethionine [27]. A significant decrease in the levels of glycerophosphoethanolamine (p = 0.02), which is a phosphatidylethanolamine catabolite, at the 72 hour time point, suggest that existing phosphatidylethanolamine may be channeled into PC synthesis reducing its rate of metabolism and further increasing flux through the PC synthesis pathway.

### Increase in Cholesterol and Sphingoid Base Production

**Figure 2:**
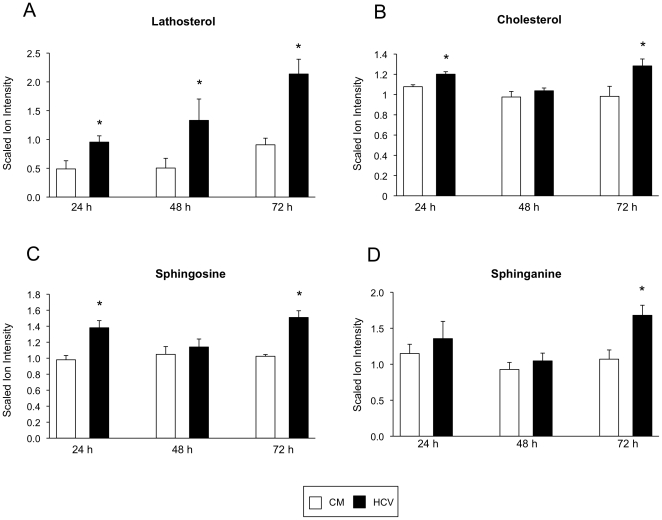
Increased cholesterol and sphingoid base production. Bar charts show the scaled ion intensity for (**A**) lathosterol, (**B**) cholesterol, (**C**) sphingosine, and (**D**) sphinganine in HCV-infected cells (black bars) and mock-infected cells (white bars) 24, 48, and 72 hours post-infection. An asterisk (*) denotes a statistically significant difference with p<0.05. Data are presented as the mean of three separate experiments with error bars representing standard deviation. CM, conditioned media.

A significant increase in the cholesterol precursor, lathosterol, was observed at all time points following infection (Figure 2A). Cholesterol levels were also significantly elevated at both the 24 h and 72 h time points (Figure 2B). A number of sphingoid bases were also affected; levels of the sphingolipid, sphingosine, were significantly elevated both 24- and 72 hours following HCV infection (Figure 2C) with sphinganine levels also significantly increased 72 hours post-infection (Figure 2D).

### Altered Fatty Acid Beta-Oxidation

**Figure 3:**
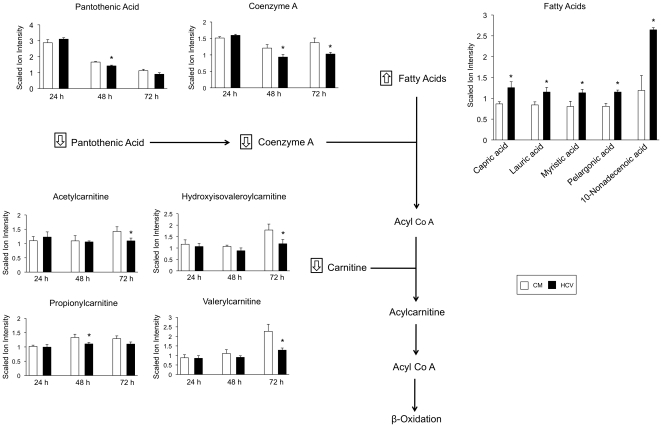
Disruption in fatty acid metabolism during HCV infection. Fatty acid oxidation pathway and bar charts showing the scaled ion intensity for coenzyme A, pantothenic acid, and carnitine derivatives in HCV-infected cells (black bars) and mock-infected cells (white bars) 24, 48, and 72 hours post-infection. Increased fatty acid concentration in HCV-infected cells 72 hours post-infection is also depicted. An asterisk (*) denotes a statistically significant difference with p<0.05. Data are presented as the mean of three separate experiments with error bars representing standard deviation. CM, conditioned media.

In HCV-infected cells, there was a significant decrease in the levels of coenzyme A (CoA), both 48- and 72-hours post-infection (p = 0.02); levels of the CoA precursor, pantothenic acid, were also significantly decreased at the 48-hour time point (p = 0.005) (Figure 3). A decrease in these metabolites suggest that activation of fatty acids, which is required for transport of fatty acids into the mitochondria for beta-oxidation, may be disrupted during HCV infection. In addition, acetylcarnitine and a number of carnitine derivatives were significantly decreased 48- and 72-hours post-infection (Figure 3). As CoA plays a vital role in the synthesis of these carnitine-conjugated metabolites, their decline may be caused by limiting CoA levels. A decrease in these carnitine conjugates may impact on the intracellular carnitine pool reducing its availability for fatty acid transport. Further evidence for a disruption in fatty acid oxidation is attributed to the fact that a number of fatty acids showed significantly increased levels at the 72-hour time point (Figure 3).

### Fluctuations in 5′-Methylthioadenosine (MTA) Metabolism

**Figure 4:**
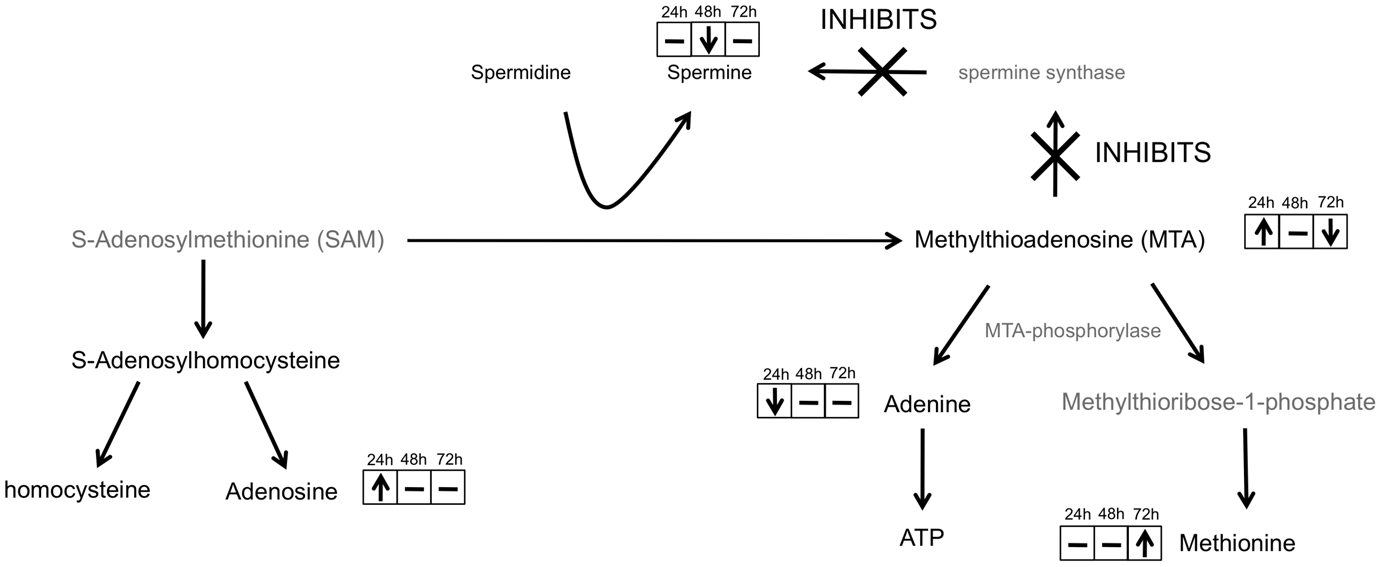
Schematic representation of MTA fluctuations during HCV infection. ‘↑’ indicates that the mean metabolite level was significantly higher (p<0.05) in HCV-infected cells compared to control. ‘↓’ indicates that the mean metabolite level was significantly lower (p<0.05) in HCV-infected cells compared to control. ‘–’ indicates that there was no change in the mean metabolite level between HCV-infected and mock-infected cells. Metabolites coloured in grey were not measured during the analysis.

MTA is a lipophilic sulfur-containing adenine nucleoside produced from S-adenosylmethionine (SAM) during the synthesis of the polyamines, spermine and spermidine [28]. MTA levels were significantly increased in HCV-infected cells at the 24-hour time point (p = 0.03); increased MTA levels have an inhibitory effect on spermine synthase, subsequently preventing the formation of spermine. This could explain the decreased spermine levels noted at the 48-hour time point (p = 0.004). Since MTA is produced as a by-product of polyamine synthesis, the decrease in spermine levels could contribute to the decreased MTA concentration observed at the 72-hour time point (p = 0.04) (Figure 4). The presence of HCV could increase MTA levels by several different methods. One of these is by upregulation of the MTA precursor SAM; evidence for this is reflected in the increased levels of SAM metabolites at the 24-hour time point - adenosine levels were significantly elevated (p = 0.008) and there was a moderate increase in s-adenosylhomocysteine concentration. (p = 0.08). In addition, SAM can methylate the amine of the phospholipid phosphatidylethanolamine yielding phosphatidylcholine; the increase in choline-conjugated lysophospholipids and decrease in phosphatidylethanolamine metabolites in our dataset, provide further evidence to suggest that SAM production may be increased. Alternatively, an increase in MTA levels may be caused by a loss in MTA-phosphorylase (MTAP) activity; MTAP is the enzyme responsible for metabolizing MTA to yield adenine and 5-methylthioribose-1-phosphate (MTR1P), which is then converted into methionine through a series of oxidation reactions. A primary consequence of the loss of MTAP activity in cellular metabolism is the intracellular accumulation of MTA and the impairment in adenine salvage to form AMP and ATP [28]. At the 24-hour time point in this study, MTA levels were significantly increased and adenine levels were significantly decreased (p = 0.04), suggesting partial MTAP activity may be lost. If this is the case, it would appear that MTAP activity is restored by the 72-hour time point as MTA levels are significantly decreased in HCV-infected cells and methionine levels are significantly increased (p = 0.03).

### Altered Redox Homeostasis

Glutathione is a key cellular anti-oxidant; a significant increase in oxidized glutathione levels (glutathione disulphide; GSSG) was noted at the 72-hour time point following HCV infection (p<0.03; Table S1), suggesting cellular redox status is disrupted. In addition, a number of gamma glutamyl dipeptides were significantly increased at all time points following HCV infection (p<0.05; Table S1), indicating there may be an increased flux through the gamma glutamyl pathway due to increased glutathione turnover. Furthermore, a significant decrease was noted in the levels of the glutathione metabolites, cysteinylglycine and s-methylglutathione at the 48 and 72 hour time points respectively (p<0.05).

## Discussion

As metabolites can directly influence cell phenotype, their quantification may depict the role of metabolic pathways in disease pathogenesis more accurately, when compared to other “omic” based analyses. It is estimated that the human metabolome contains in the range of 2,000–3,000 metabolites [20]; this reduced complexity in metabolomic data facilitates the detection of meaningful biological changes. In this study, a metabolomics-based approach was used to compare global biochemical profiles between cultured HCV-infected and uninfected hepatocytes. The results obtained highlight some previously unreported metabolic interactions and provide greater insight into the effect HCV has on host-cell biochemical processes.

During early HCV infection, there was evidence of increased flux in a number of biosynthetic pathways including the pentose phosphate pathway, RNA synthesis and amino acid metabolism. In cell culture, elevated amino acid levels typically reflect increased uptake from the media in response to anabolic and energy demands. Evidence for an upregulation in biosynthetic pathways during HCV infection has been demonstrated previously; high levels of CTP and UTP are critical parameters for efficient HCV replication [29], and a recent proteomics-based study reported upregulation of a number of enzymes involved in nucleotide synthesis and homeostasis during HCV infection [14]. The elevated metabolism and improved energy status observed in this study are likely to occur in response to the increased metabolic demand resulting from viral replication.

A number of phosphatidylinositol and phosphocholine based LPLs were significantly increased at all time points examined following HCV infection. Phosphatidylinositols have previously been implicated in HCV infection – HCV NS5A has been shown to interact with the phosphoinositide 3-kinase pathway [30] and phosphatidylinositol 4-kinase is essential for efficient HCV replication [31]. In recent times, LPLs have become the focus of many studies since it was discovered that, aside from their involvement in phospholipid metabolism, they play a role in signal transduction [26]. They have also been implicated in a wide range of disorders such as inflammation, autoimmune disease, and cancer [26], [32]. Lysophosphatidylcholine elicits various proinflammatory effects including up-regulation of adhesion molecules and increased endothelial permeability [33]. An ongoing accumulation of lysophospholipids during HCV infection could play a role in subsequent liver damage caused by persistent hepatic inflammation. Lysophospholipids generated by phospholipase A_2_ have been shown to modulate plamsa membrane structure and assist in the budding of raft-associated plasma membrane particles. The addition of phospholipase A_2_ inhibitors to an influenza A virus-infected cell culture, resulted in a reduction in viral budding and decreased viral titres [34]. It is possible that lysophospholipids may play a role in lipid raft formation and assist in the budding of HCV. A significant increase in a number of intermediates involved in phosphatidylcholine synthesis was also noted at different time points during HCV infection. The importance of the PC biosynthetic pathway in HCV replication has been shown previously; one recent study demonstrated a progressive accumulation of several PC species during HCV infection [14]. In addition, Yao *et al* demonstrated that the enzyme, long chain acyl-CoA synthetase 3 (ACSL3), is specifically required for incorporation of fatty acids into phosphatidylcholine and treatment of cells with siRNA targeting ACSL3 inhibited the secretion of VLDL and HCV particles from hepatocytes [35]. PC synthesis is required for the normal secretion of VLDL by hepatocytes [27] and it has previously been suggested that HCV uses the VLDL assembly and secretion pathway for maturation and secretion of viral particles [10], [11]. This study provides further support for the importance of PC during HCV replication; it is possible there is an increased flux through the PC synthesis pathway in HCV-infected cells in order to enhance lipoprotein production and aid in viral secretion.

A significant increase in cholesterol and a number of sphingoid bases was noted at various time points during HCV infection. Another recent study also found increased cholesterol levels in HCV-infected Huh-7.5 cells [19] and it has previously been demonstrated that elements of the cholesterol and sphingolipid biosynthetic pathways are necessary for HCV replication; treatment of HCV-infected cells with cholesterol lowering drugs can inhibit HCV RNA replication [6], [36] and inhibitors of the sphingolipid biosynthetic pathway are capable of blocking HCV virion production [12]. During HCV replication, a membranous web, containing viral and host proteins, is formed and localizes inside what are known as lipid rafts. These rafts are small, cholesterol and sphingolipid-enriched domains that have been implicated in protecting the HCV replication complex against degradation [37]. In addition, a recent study has shown that virion-associated cholesterol and sphingolipid are critical for virion maturation and infectivity [12]. It is possible that cholesterol and sphingolipids are increased within HCV-infected cells in order to enhance lipid raft production and support virion formation. As well as playing a structural role in membrane formation, sphingolipids also act as second messengers in transmembrane and intracellular signal transduction pathways [38]. Both sphingosine and sphinganine have been shown to play a role in apoptosis induction [39]. Sphingosine is involved in caspase activation and is also capable of inhibiting the anti-apoptotic molecule, Akt [38]. An increase in free sphingoid bases during HCV infection may contribute to HCV-associated liver disease.

An increase in fatty acid concentration and a decrease in mediators of fatty acid transport suggest fatty acid oxidation may be disrupted during HCV infection. Fatty acids play an important role in HCV replication; a previous study has shown that saturated fatty acids are required for HCV replication, with myristic and lauric acid capable of enhancing viral replication [36]. Interestingly, 5 of the 6 fatty acids that were observed to increase after HCV infection in the current study, including myristic and lauric acid, were saturated. Evidence for impaired degradation of lipids has been reported previously; a recent genomics-based study reported a significant decrease in a number of genes involved in the degradation and oxidation of fatty acids in HCV infected cells [40]. In addition, HCV core expression has been shown to contribute to hepatic accumulation of lipids in mice, and hepatic downregulation of fatty acid metabolism-associated genes have been reported previously in HCV-infected patients [6]. The results of the current study suggest additional routes through which fatty acid degradation can be perturbed; it is conceivable that HCV has an inhibitory effect on the CoA and carnitine metabolic pathways limiting the availability of these metabolites, thereby disrupting fatty acid activation and mitochondrial transport.

This study reports for the first time the involvement of MTA in HCV infection. Previous studies have shown that increased levels of MTA exhibit strong anti-oxidant effects, reducing liver damage and fibrosis [28], [41]. In addition, MTA has proved to be a powerful modulator of inflammation in vivo [42], [43] and has shown potential as a treatment for both chronic liver disease and viral infections [41], [42], [44]. It is possible that MTA levels are increased within HCV-infected cells immediately after infection, in order to play a hepatoprotective role. High concentrations of MTA have been shown to reduce the growth rate of cells [28] and can cause apoptosis in human hepatocytes [45]; this could negatively impact HCV replication so its possible that HCV interacts with SAM and/or MTAP expression, increasing MTA breakdown, explaining the significant decline in MTA levels at the 72-hour time point. Persistently low levels of MTA could contribute to the chronic inflammation and liver damage associated with HCV infection.

HCV infection has long been associated with an increase in markers of oxidative stress, many of which play a role in HCV-associated liver damage [46]. An increase in the levels of gamma glutamyl dipeptides in HCV infected cells provides evidence of an increased flux through the glutathione pathway. A recent study reported an increase in serum gamma glutamyl dipeptides for a number of liver diseases, including HCV. Furthermore, they also demonstrated that synthesis of these dipeptides correlate with the levels of hepatic GSH production, suggesting they can provide valuable information on the hepatic redox state [47]. The increased GSSG levels noted in the current study provide further support for the induction of oxidative stress within HCV-infected cells. Furthermore, a significant decrease in the levels of glutathione metabolites, s-methylglutathione and cysteinylglycine, suggest there may be a reduced capacity to maintain intracellular redox homeostasis. GSSG levels have previously been associated with loss of mitochondrial integrity and caspase-activation [48]. In an effort to negate the detrimental effects of oxidative stress incurred by HCV infection, it is possible the increased GSSG levels play a role in subsequent liver damage through the induction of apoptotic pathways.

A limitation of the current study is the absence of comparative proteomic and/or transcriptomic data. However, the results obtained correlate well with previously reported proteomic/genomic-based studies. It should also be noted that the Huh-7.5 cells used in the study are hepatoma cells that differ in several aspects from primary human hepatocytes. As cells were infected using a genotype 2a strain, it would be interesting to compare and confirm the current findings using HCV cell culture systems for other major genotypes.

This study is unique in that it is the first comprehensive report documenting metabolite fluctuations in HCV-infected hepatocytes. Whilst confirming the results of previous genomic/proteomic based reports, the current study also provides previously unreported findings on the complex interactions between HCV and various metabolic pathways. We describe for the first time the involvement of LPLs and MTA in HCV infection and provide further information on the impaired degradation of fatty acids. We also describe alterations in the levels of glutathione disulphide, saturated fatty acids and sphingoid bases, highlighting additional mechanisms through which the histopathological manifestations of HCV may originate. The descriptive nature of these findings warrant subsequent analysis; future studies, which are currently in progress in our laboratory, should focus on investigating the affected pathways in greater detail in order to fully elucidate the biochemical significance of these findings.

## Supporting Information

Table S1 List of the 250 metabolites quantified in HCV-infected Huh-7.5 Cells. Metabolite levels were quantified 24, 48, and 72, hours post-infection. Significantly increased metabolites are highlighted in red with decreased metabolites highlighted in green.
(XLS)

Table S2 Comparative analysis of metabolite levels between time points for both un-infected and HCV-infected Huh-7.5 cells. Significantly increased metabolites are highlighted in red with decreased metabolites highlighted in green.
(XLS)
